# Measurement for gap balancing technique in patients undergoing total knee arthroplasty: a large retrospective observational study

**DOI:** 10.1186/s13018-022-03104-6

**Published:** 2022-04-07

**Authors:** Zhao Xuequan, Zhao Bin, Yao Shuzhang, Cao Kanduo, Ren Chongxi

**Affiliations:** 1 Department of Orthopaedics, Cangzhou Clinical College of Integrated Traditional Chinese and Western Medicine of Hebei Medical University, Qian Tong North Street No. 17, Cangzhou City, 061000 Hebei Province China; 2 Department of Oncology Surgery, Cangzhou Clinical College of Integrated Traditional Chinese and Western Medicine of Hebei Medical University, Cangzhou, 061000 China

**Keywords:** Total knee arthroplasty, Osteotomy location, Gap-balanced, Measuring method

## Abstract

**Background:**

Many traditional methods are available to prevent unbalance of extension and flexion gap during total knee arthroplasty (TKA), but there are no reports on the use of measurement and positioning method before tibial osteotomy with self-made tools. We designed a self-made tool measuring the location before tibial osteotomy and determined the clinical effect.

**Methods:**

The retrospective study included patients who received TKA at our hospital, between January 1, 2012 and December 31, 2015. A new method, named as the measurement and localization before osteotomy with self-made tools, was developed to measure the osteotomy position of the posterior femoral condyle during TKA. They were divided into two groups, one that received the new method (Group I), and the other that received the traditional method as a control (Group I I). HSS score, Oxford score, VAS score and knee joint activity were evaluated in two groups.

**Results:**

One hundred and eighty-seven of 210 eligible patients were included. The function of knee joint in all patients was improved and the pain was obviously relieved. Significant differences were found in the HSS score, Oxford score, VAS score, knee joint activity between two groups at 5-year follow-up (*p* < 0.05).

**Conclusions:**

The biomet knee prosthesis was selected for all intraoperative implants. All operations were completed by the same senior surgeon. The use of self-made tools may contribute to improve the balance between flexion and extension gaps as well as the balance between internal and external gaps during TKA, and overcome knee flexion instability.

## Background

Total knee arthroplasty (TKA) is one of the most effective surgical treatments for pain relief and functional recovery in patients with advanced degenerative osteoarthritis or rheumatoid arthritis [1–3]. Axial alignment of the lower limb correction is a key step during TKA, which can increase patient satisfaction and prolong the use of the prosthesis. With the aging population and the prevalence of obesity, the number of patients receiving TKA has increased rapidly in the past two decades, and TKA has now become one of the most common and costly medical procedures in the USA and Canada [4, 5]. Although the studies reports that the survivorship and surgeon-based measures rates after TKA is as high as 80% [6], these measures do not account for limb alignment following TKA such that chronic pain for poor limb alignment remains a major health burden for many patients.

A good axial alignment of the lower limb can make patients get closer to the normal way of the knee joint movement, avoiding early failure caused by uneven loading of the prosthesis and bone cement and keeping the function of the knee joint [7–9]. The trajectories of patellofemoral joint and knee joint stability will be affected when the femoral or tibial prosthesis is not suitable for rotational insertion [7]. It can lead to complications such as anterior knee pain, abnormal gait and loosening of the prosthesis.

Here we introduced a new method which measured the osteotomy location during TKA and achieved satisfactory imaging effect and clinical results.

## Methods

### Study cohort

We retrospectively reviewed the medical records of patients who underwent TKA by given a new measuring method of osteotomy from January 1, 2012 to December 31, 2015. Patients were included in the analysis if they met the following criteria: (1) identified preoperative diagnosis of osteoarthritis; (2) performed preoperational assessment manually by the orthopedist, such as the varus deformity Angle ≤ 15°; (3) had follow-up of more than 5 years. Patients who underwent TKA for other diseases of the knee joint were excluded. The characteristics of the patients are listed in Table 1.

**Table 1:** Patients characteristics

	Group I (n = 43)	Group II (n = 48)	*P* value
Age^a^, years	62.4 ± 4.2	61.3 ± 4.8	0.183^†^
Sex			
Male	22	23	0.461^‡^
Female	21	25	
Affected side			
Right	25	29	0.497^‡^
Left	18	19	
Body Mass Index^a^, kg/m^2^	23.0 ± 1.8	22.5 ± 1.7	0.306^†^
Follow-up period^a^, months	36.3 ± 5.8	37.1 ± 6.9	0.565^†^
Interval between morbidity and surgery^a^, years	3.7 ± 3.0	3.9 ± 2.9	0.756^†^

^a^The values are given as the mean and the standard deviation

^†^Student t-test; *p* < 0.05 demonstrates significance

^‡^Fisher exact test; *p* < 0.05 demonstrates significance

The patients in the study were divided into two groups, one that received the new method (Group I), and the other that received the traditional method as a control (Group II). Preoperative examinations (hematology and imaging tests) were should be completed and surgical contraindications were ruled out.

The study was approved by the local Clinical Research Ethics Committees (NO.***), and written informed consent was waived due to the retrospective nature of the study.

### Surgical procedure

The patient in Group I was in a supine position. After the successful implementation of subarachnoid anesthesia or combined epidural anesthesia, a parapatellar medial approach was used to dissect and expose the distal femur, and the femoral bone marrow cavity was drilled with bone bur. By an appropriate eversion angle control module, fixing the distal femoral osteotomy module with a fixed needle, and confirming the osteotomy position with the osteotomy measurement device, the distal femoral osteotomy was performed. An assembled tibial extramedullary positioning device was installed on the lower leg to determine the vertical force line, rotation force line and the backward tilt angle of the positioning rod; the tibial osteotomy module was fixed on the tibia with a fixing needle (following the natural tibial bow), and the proximal tibial osteotomy was performed after being reconfirmed by a bone plane measuring device.

**Fig. 1:**
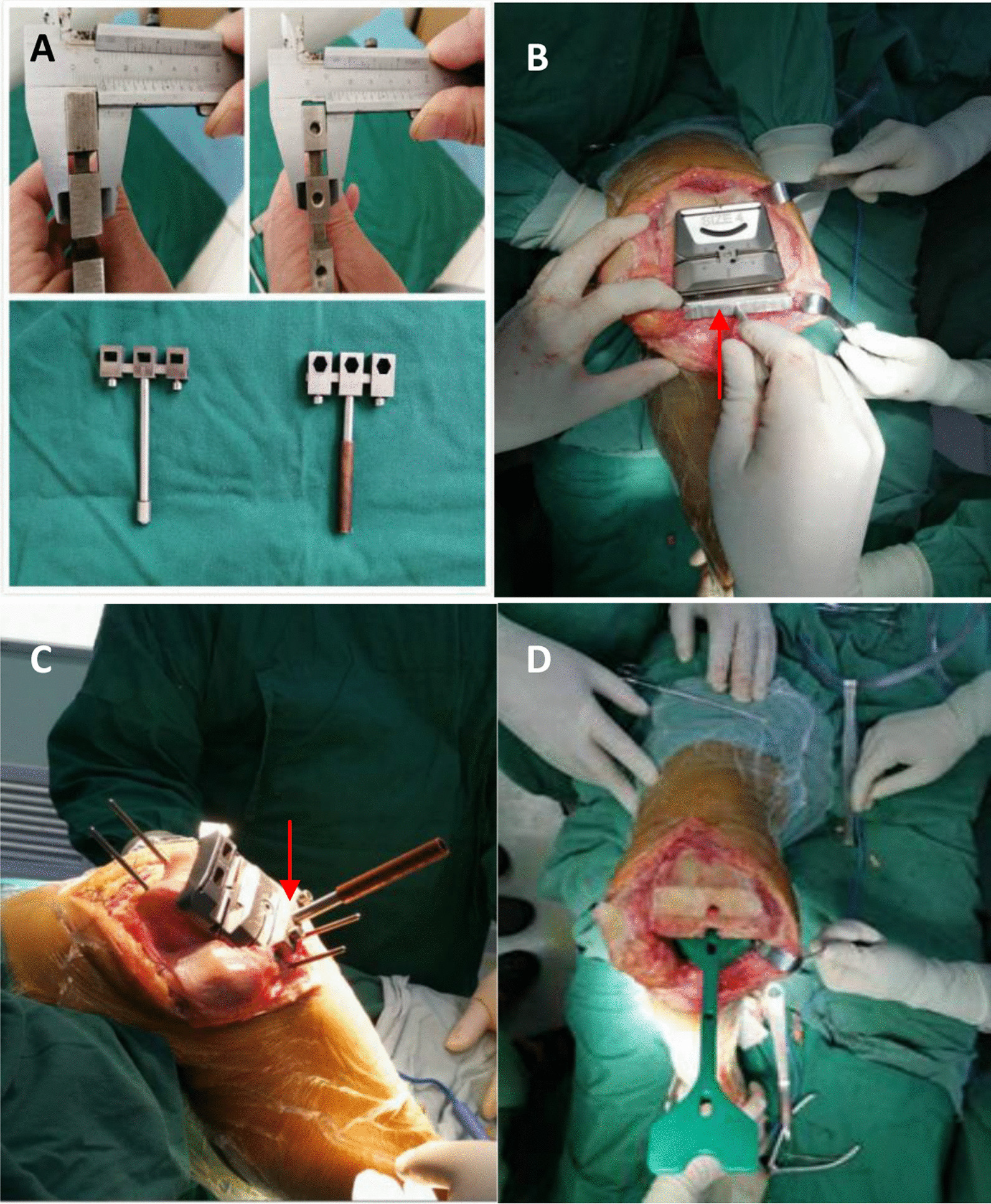
**A**–**D** New measurement of osteotomy location: self-made tools are used to adjust the flexion gap (red arrow). **A** Self-made tools which were measured for thickness. **B** Self-made tool was used intraoperatively. **C** The osteotomy location was measured intraoperatively. **D** The method achieved the balance of extension and flexion gap

The extension gap of the affected knee joint was measured intraoperatively, and the measured value was recorded as A (mm). Attaching a femur size gauge to the distal femur osteotomy section, and the posterior two wings hook the posterior medial and lateral condyle of the femur (rotation angle is 3° by default). Moving the measuring hook on the device to the highest point of the anterior cortex of the femur, the size of the anterior and posterior osteotomy module was determined according to the measured value; and the appropriate four-in-one osteotomy plate was used (the osteotomy line of the posterior femoral condyle is basically parallel to the lower edge of the osteotomy plate). The measured value between the lower edge of the osteotomy plate and the osteotomy line of the posterior femoral condyle was recorded as B (mm). A self-made osteotomy module (Fig. 1, red arrow) with thickness of C (mm) was placed at the flexion position of the affected knee at about 90°, so that the lower edge osteotomy line was parallel to the plane of the osteotomy module, resulting in A = B + C, and its extension and flex gaps were balanced.

*Notes* The extension gap A was used as the standard value and measured at the 90°flexion position so that the osteotomy line at the lower edge of the four-in-one osteotomy plate was parallel to the tibial plane. When the gap is unequal (that is, A < B + C or A > B + C), performer can adjust the module to move the knob up and down to make A = B + C. When the adjustment range exceeds 2 mm, orthopedist should consider readjusting the distal femur osteotomy or replace the four-in-one osteotomy module of the femur. Four osteotomy modules with a thickness of 10–14 mm were designed. It was used according to the specific situation during the operation.

Traditional measurement was used in the TKA operation in Group II.

### Postoperative rehabilitation

After the surgery, all patients were required to perform ankle joint exercises, and the scope of motion was encouraged within tolerable range. One day after the surgery, isometric quadriceps, active ankle and straight-leg raise exercises were commenced. Toe-touch weightbearing was initiated immediately after the surgery, and partial weightbearing was allowed at 4 days after the surgery for the next 4 weeks. At 12 weeks, the walking aid was removed.

### Statistical analysis

Primary and secondary end points were compared between the two groups. The Student test or the Fisher exact test was used to analyze continuous variables. Multiple comparisons were performed with repeated-measures analysis of variance (ANOVA). All computations were performed with standard software (SPSS version 22.0 for Windows; IBM), with significance set at *p* < 0.05.

## Results

### Patient characteristics

There were no significant differences between the treatment groups in terms of demographic characteristics or baseline outcome measures (Table 1).

### Outcomes

**Fig. 2:**
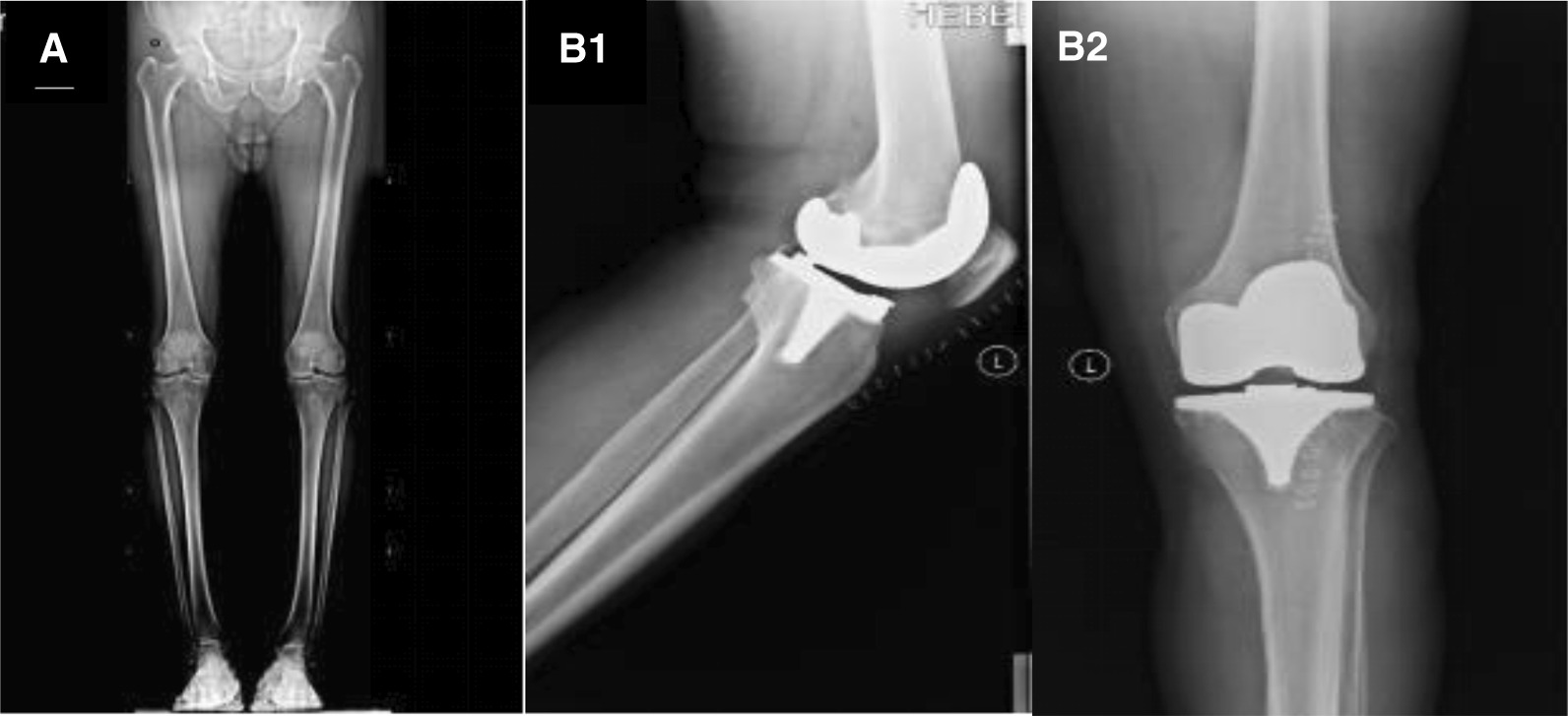
**A**, **B** X-ray findings before and after surgery. **A** Preoperative X—ray showed the knee osteoarthritis. **B** Positive X-ray (**B1**) and lateral X-ray (**B2**) examination at 1 day after operation showed the image performance after knee replacement

We enrolled 210 patients in the trial. Ten patients died after 2-year of follow-up, outpatient follow-up failed in eight cases, and five case was amputated due to lower limb tumor. Finally, a total of 187 patients met the study criteria and were included in the analysis. After these patients underwent TKA with this measurement method, the knee joint function was improved and the pain was significantly relieved. In addition, the knee joint deformity was corrected. The postoperative imaging examination was satisfactory (Fig. 2). None of the 187 patients had failed surgery. All patients obtained the balance of extension and flexion gap and inner and outer gap after operation without medium-term instability of bended knee joint.

The difference between the two groups in the amount of intraoperative blood loss and tourniquet duration were not statistically significant(*p* > 0.05).The preoperative HSS score, Oxford score and VAS score, knee joint activity also demonstrated no statistical differences among the groups before the operation (*p* > 0.05). The mean postoperative after 5 years of HSS scores were 86.7 and 74.5 in group I and group II, respectively, and the difference among the groups showed statistical significance (*p* = 0.000); The mean of oxford scores after 5 years were 19.4 and 21.9 in group I and group II , respectively, and the difference among the groups showed statistical significance (*p* = 0.000); The mean of VAS scores after 5 years were 2.6 and 3.8 in group I and group II, respectively, and the difference among the groups showed statistical significance (*p* = 0.000). The mean of knee joint activities after 5 years were 91.9 and 88.8 in group I and group II, respectively, and the difference among the groups showed statistical significance (Table 2).

**Table 2:** Evaluation of clinical indicators

	The number of cases^a^		HSS^b^		Oxford^b^		VAS^b^		Knee joint activity^c^	
	Group I	Group II	Group I	Group II	Group I	Group II	Group I	Group II	Group I	Group II
Preoperative	43	48	66.8 ± 5.8	65.9 ± 7.9^(1^	42.1 ± 7.9	40.7 ± 6.9^(1^	7.3 ± 0.8	7.6 ± 0.8^(1^	77.6 ± 4.0	78.2 ± 4.3^(1^
24-month follow-up			84.5 ± 9.6	73.9 ± 12.7^(2^	19.9 ± 2.8	23.0 ± 1.4^(2^	2.7 ± 0.9	4.5 ± 1.2^(2^	92.4 ± 5.3	85.5 ± 4.3^(2^
*t* value			0.574^(1^/4.141^(2^		1.118^(1^/6.426^(2^		1.820^(1^/8.348^(2^		1.373^(1^/7.011^(2^	
*P* value			0.569^(1^/0.000^(2^		0.270^(1^/0.000^(2^		0.075^(1^/0.000^(2^		0.177^(1^/0.000^(2^	

^a^Number(n)

^b^Score

^c^°

## Discussion

**Fig. 3:**
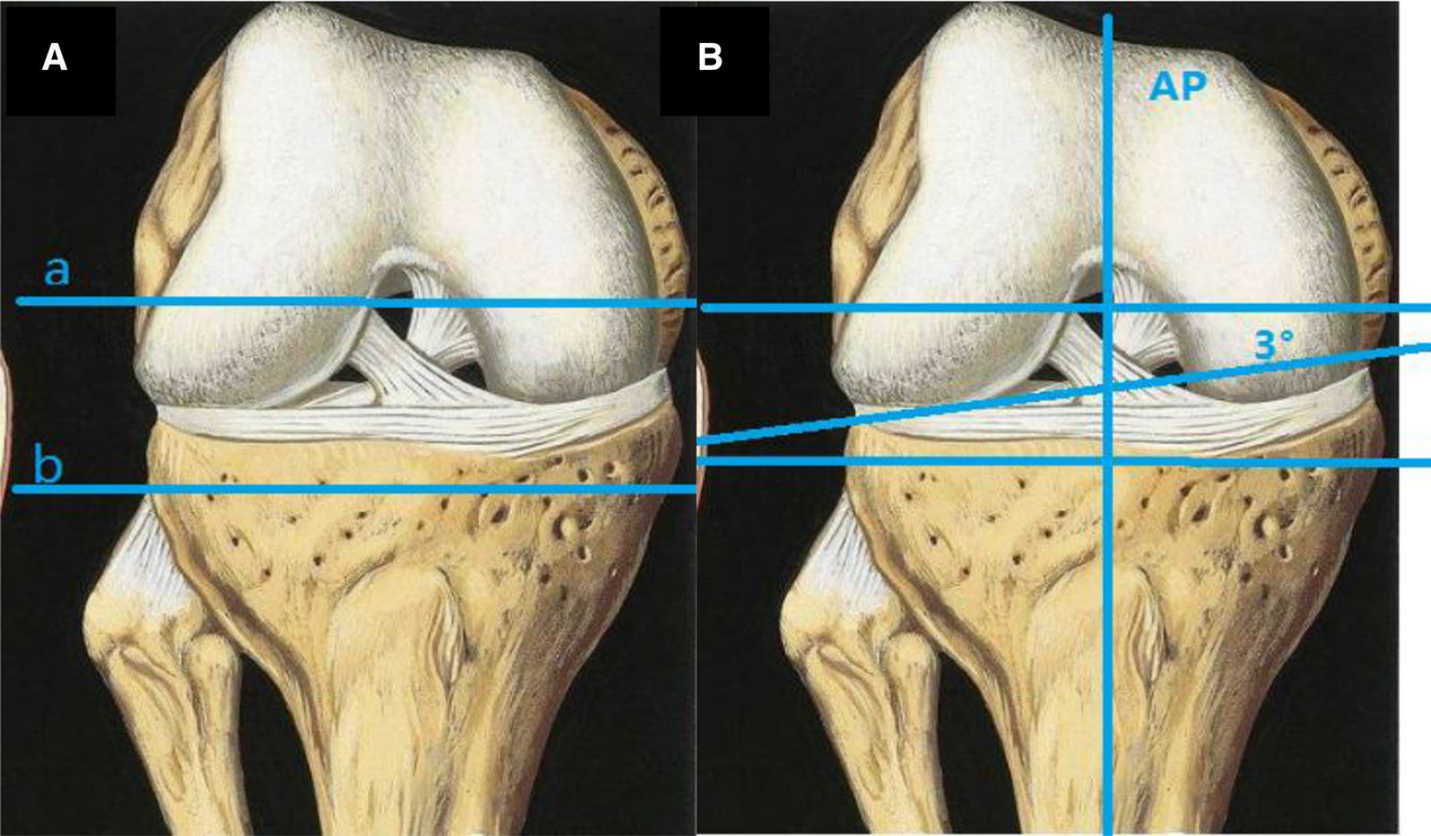
**A**, **B** Osteotomy of posterior femoral condyle in total knee arthroplasty. **A** Gap balancing technique: with tibial plateau as the benchmark, the "buckling gap" of the rectangle was truncated by gravity, distraction and other methods. **B** Measuring osteotomy technique: posterior femoral condyle osteotomy was performed perpendicular to the AP axis or at a 3° Angle with the connecting line of the posterior femoral condyle

Obtaining satisfactory stability in the coronal plane is critical for the long-term success of TKA and requires precise rotational alignment of the femoral component. Malrotation of the femoral component has been associated with numerous undesirable conditions including patellofemoral and tibiofemoral instability, arthrofibrosis, knee pain, and disturbed knee kinematics [8–10]. Many utilize a measured resection technique to search correct femoral component rotation and subsequent coronal plane stability. There are two methods for osteotomy of posterior femoral condyle in flexion gap during total knee arthroplasty: gap balance and osteotomy measurement [11]. The measurement of osteotomy is usually taken by osteotomy of the posterior femoral condyle based on 3°external rotation on the coronal plane of the distal femur (Fig. 3), but there is a large individual difference in the rotation angle [12–14]. In the measurement of osteotomy, there are also osteotomy methods marked by intraoperative surgical touch of the femoral epicondylar axis. However, not a few literatures reported that the internal epicondylar groove can only be identified in some patients, and the occurrence rate of patients with severe osteoarthritis is less, and the accuracy of intraoperative positioning of the transfemoral epicondylar axis is poor. As a result, the accuracy of intraoperative osteotomy mostly depends on the experience of the operator [15]. Intraoperatively, it is often the case that accurate distal femur osteotomy is difficult due to the formation of an uneasily adjusted flexion gap. As our recommendation, this new osteotomy position measurement avoids an imbalance of the flexion and extension by measuring the accurate osteotomy gap at the distal femur.

Many studies [15–18] have reported the "osteotomy gap measurement technique" used in TKA procedures. It is generally believed in the orthopedic community that TKA surgery should be aimed at obtaining the same rectangular gap in knee flexion and extension, and achieving soft tissue balance before osteotomy is the optimized choice for the best surgical results. However, traditional operation techniques rely on soft tissue balance to achieve gap balance after osteotomy. During the operation, the osteotomy is completed by referring to the anatomical landmarks; once the gap is found to be unbalanced, the soft tissue release is continued, that is, first osteotomy and then release. Since the loosening of soft tissue increases the flexion gap and the extension gap by different amounts, it is difficult to achieve an accurate balance between the flexion gap and the extension gap at the same time. It is based on this that we introduced and used the self-made measurement module to accurately measure the flexion–extension gap before osteotomy in TKA procedures. The advantage of the new measurement of osteotomy location was the ability to measure the flexion gap before osteotomy of the distal femur, and the amount of osteotomy was determined according to the individual differences of patients during TKA.

The gap balance technique strictly complies with the flexion and extension gap balance standard, and maximally meets the requirements of the vertical osteotomy surface of the distal femur and the lower limb alignment. In patients with relatively mild knee joint disease, both the measurement osteotomy technique and the gap balance technique can achieve satisfactory clinical results. However, for patients with severe knee joint disease and dysplasia, the commonly used anatomical landmarks are often difficult to identify, there are many hyperostotic osteophytes, and ligament degeneration or variation exists. Simply using the measurement osteotomy technique may cause osteotomy positioning errors. However, gap balancing techniques have the potential to improve surgical precision and reduce unnecessary surgical trauma. Gap balancing techniques give priority to flexion–extension gap balancing. In patients with complex deformity of knee osteoarthritis, if the osteophyte is not properly handled and the ligament contractures or laxity, the femoral osteotomy is directly performed, and it is easy to have too much or too little osteotomy of the posterior femoral condyle. If the proximal tibia is varus or valgus osteotomy, the medial flexion gap will become larger or smaller, resulting in too little or too much femoral posteromedial osteotomy, which further leads to abnormal rotation of the femoral component. Better flexion gap and femoral prosthesis rotational alignment can be obtained by using gap balance technology, which not only reduces unnecessary soft tissue dissociation, but also shortens soft tissue trauma and operation time, which is beneficial to the recovery of knee joint function. Cristian Aletto et al. [19] investigated the functional outcomes of computer navigated total knee arthroplasty in a series of 180 patients (200 knees), reporting that computer-assisted TKA allows reproducible alignment and kinematics, reducing outliers, provides ligament balancing and ensures good short-term outcomes. Stefano Marco Paolo Rossi et al. [20].used the tensor as an extramedullary cutting guide for the distal femoral cut based on a 90° tibial resection, demonstrated satisfactory encouraging clinical outcomes at mid-term follow-up leaving a residual deformity on the coronal plane. Benazzo et al. [21]. used a new design with a progressive increased keel medialization according to the size was implanted and followed up for seven years, demonstrated an excellent outcome of this design.

We believe that the poor rotation axis of prosthesis by osteotomy is related to the individual difference of posterior condyle angle in the population. Therefore, the rotation angle of the osteotomy plate should take into account the individual differences of the femur. During TKA, equal rectangular space should be obtained when the knee joint is extended and flexed. Release the medial ligament in patients with varus and release the lateral ligament in patients with ectropion. The goal is to obtain the same rectangular gap regardless of the osteotomy method. The new technique uses the self-made gap gasket to measure the flexion gap before osteotomy of femur, so that the imbalance of postoperative space was avoided. The orthopedist can adjust the position of the osteotomy plate to improve the balance of extension and flexion gap according to the gap measured by the self-made gasket. However, this trial is a single-center retrospective study, lacks imaging assessment data, and is difficult to discuss further, such as the lack of analysis of femoral angle, tibial angle, knee valgus angle, femoral prosthesis flexion angle and joint space separation variables. A multicenter, prospective, multi-angle and dynamic study is needed to further improve the application value of imaging assessment in the guidance of total knee arthroplasty osteotomy.

## Data Availability

The datasets used and analyzed during the present study are available from the corresponding author on reasonable request.

## Abbreviation

TKA: Total knee arthroplasty
